# The Cincinnati incision is safe and effective for revision surgery for insertional tendinopathy of the Achilles tendon

**DOI:** 10.1038/s41598-022-10730-x

**Published:** 2022-04-22

**Authors:** Nicola Maffulli, Nikolaos Gougoulias, Gayle D. Maffulli, Francesco Oliva, Filippo Migliorini

**Affiliations:** 1 Department of Medicine, Surgery and Dentistry, University of Salerno, Via S. Allende, 84081 Baronissi, SA Italy; 2 School of Pharmacy and Bioengineering, Keele University School of Medicine, Thornburrow Drive, Stoke on Trent, England; 3 Barts and the London School of Medicine and Dentistry, Centre for Sports and Exercise Medicine, Queen Mary University of London, Mile End Hospital, 275 Bancroft Road, London, E1 4DG England; 4 Foot Ankle Clinic. Iaso Thessalias Hospital, 41500 Larisa, Greece; 5 Frimley Park Hospital, Frimley, GU16 7UJ Surrey UK; 6 Wholelife Clinics, London, UK; 7 Department of Orthopaedic, Trauma, and Reconstructive Surgery, RWTH Aachen University Hospital, Pauwelsstraße 30, 52074 Aachen, Germany

**Keywords:** Medical research, Outcomes research

## Abstract

The present study reports the outcomes of revision surgery using a Cincinnati incision with tendon debridement and calcaneoplasty for insertional Achilles tendinopathy (IAT) in a cohort of patients at 24-month follow-up. Patients undergoing revision surgery following failed previous surgery for IAT were prospectively recruited. Patients were assessed pre-operatively and at 3, 6,12 and 24 months. The Victorian Institute of Sport Assessment Scale for Achilles Tendinopathy (VISA-A), the EQ5D questionnaire and the visual analogue scale (VAS) were used for evaluation. Data from 33 patients with a mean age of 43.9 years old are reported. 27% (9 of 33 patients) were female. The left side was involved in 58% (19/33) of patients. No clinically relevant complications were reported in any of the patients. Most of subscales of EQ5D improved at last follow-up: Usual Activities (*P* = 0.01), Mobility (*P* = 0.03), Pain/Discomfort (*P* = 0.001), Thermometer (*P* = 0.04). No statistically significant change for the subscales Self-Care (*P* = 0.08) and Anxiety-Depression (*P* = 0.1) was evidenced. The VISA-A score improved significantly at last follow-up (*P* < 0.0001), as did the VAS score (*P* < 0.0001). These results indicated that a Cincinnati incision followed by tendon debridement and calcaneoplasty for revision surgery for IAT is feasible and reliable, achieving clinically relevant improvement in the VISA-A, EQ5D and VAS at 24 months follow-up.

## Introduction

Achilles tendinopathy is a common musculoskeletal condition. Approximately 6% of the general population suffer from Achilles tendinopathy during their life time^1^. The term tendinopathy is a generic descriptor of the clinical conditions arising from overuse in and around tendons^2^. Achilles tendinopathy are divided into midportion and insertional ailments^3^. The latter accounts up to 24% of Achilles tendinopathy presentations^4^. Insertional Achilles tendinopathy (IAT) affect mainly adult individuals, retired athletes, especially those with lower limb imbalances, higher body mass index (BMI) or those who initiate unaccustomed physical activity^5^. IAT symptoms are greater overnight and with restart of the physical exercise^5^. Accurate imaging investigation is recommended for the diagnosis and follow-up evaluation^6,7^. The diagnosis of IAT is not always straight forward, and pitfalls are common. The surrounding structures of the Achilles tendon can become inflamed and injured, mimicking IAT. Magnetic resonance and ultrasonography have high sensitivity and specificity for IAT^8^. Conservative modalities are usually advocated as first line treatment in patients with IAT^9–11^. In IAT patients whose symptoms are not alleviated by conservative management, surgery is indicated^12,13^. Several surgical procedures for refractory IAT have been described^14–16^. However, regardless to the surgical intervention, up to 20% of patients experience poor outcomes^17–19^. The management of those patients with IAT in whom symptoms do not respond to surgery is problematic, with limited options and unpredictable results. Moreover, current evidence concerning the management and outcomes of those patients are lacking. Surgical debridement through a transverse Cincinnati incision has been successfully used for primary IAT^20^. The Cincinnati incision allows to expose the whole of the insertion area of the Achilles tendon, with full access to the posterior aspect of the calcaneus, and the regions medially and laterally to it. In addition, a Cincinnati incision lies along the Langer's lines, and thus is not subjected to the skin healing problems typical of the other incisions in this area. To the best of our knowledges, no study investigated the outcome of revision surgery in patients who continued to report symptoms of IAT following surgery. Thus, the purpose of the present study was to report the outcomes of surgical debridement and calcaneoplasty using a Cincinnati incision employed in revision procedures for failed surgery for IAT at midterm follow-up. We hypothesized that this technique can be also safely employed in revision settings for IAT with an improvement of patient reported outcome measures (PROMs).

## Material and methods

### Study protocol

The present study was conducted according to the Strengthening the Reporting of Observational Studies in Epidemiology: the STROBE Statement^21^. All procedures reported in the present investigation were approved by the Ethics Committee of the University of Salerno, Italy (CESa 01,252,009/Rev2). Patients undergoing revision surgery following failed index surgery for IAT were prospectively recruited at the University of Salerno, Italy in the period 2013 to 2017. The surgical indications were: (1) symptomatic chronic non-calcified IAT; (2) previous failed surgical treatments;(3) imaging evidence (radiography, magnetic resonance and/ or ultrasonography) suggestive of IAT (e.g. residual Haglund deformity, imaging signs of abnormalities of the insertion portion of the Achilles tendon); (4) no response to at least 6 months of conservative treatment. Exclusion criteria were: (1) pregnancy or lactation; (2) any uncontrolled chronic or acute disease; (3) malignancy; (4) bilateral involvement; (5) metabolic disorders.

### Surgical technique

**Figure 1:**
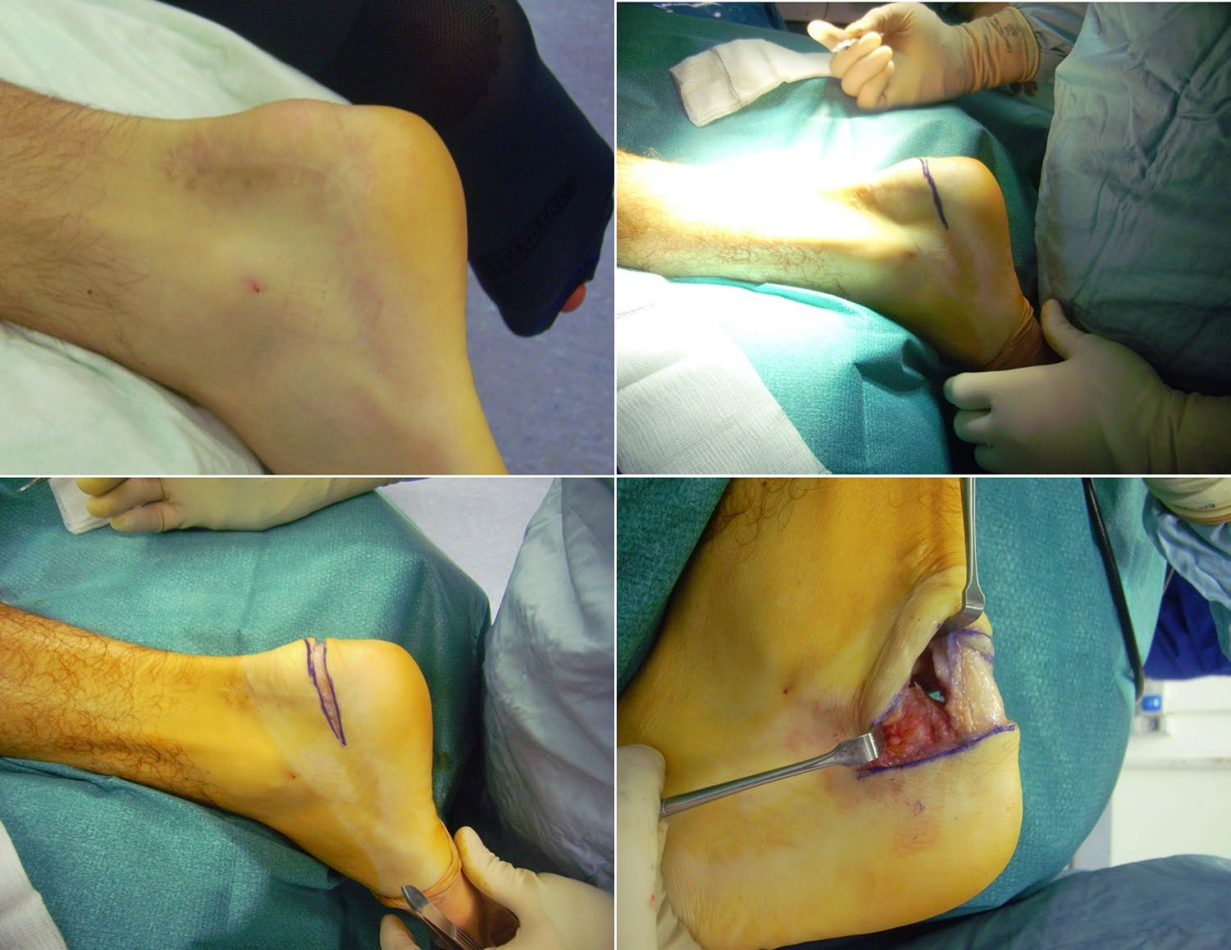
Failed lateral open approach revised with Cincinnati incision.

**Figure 2:**
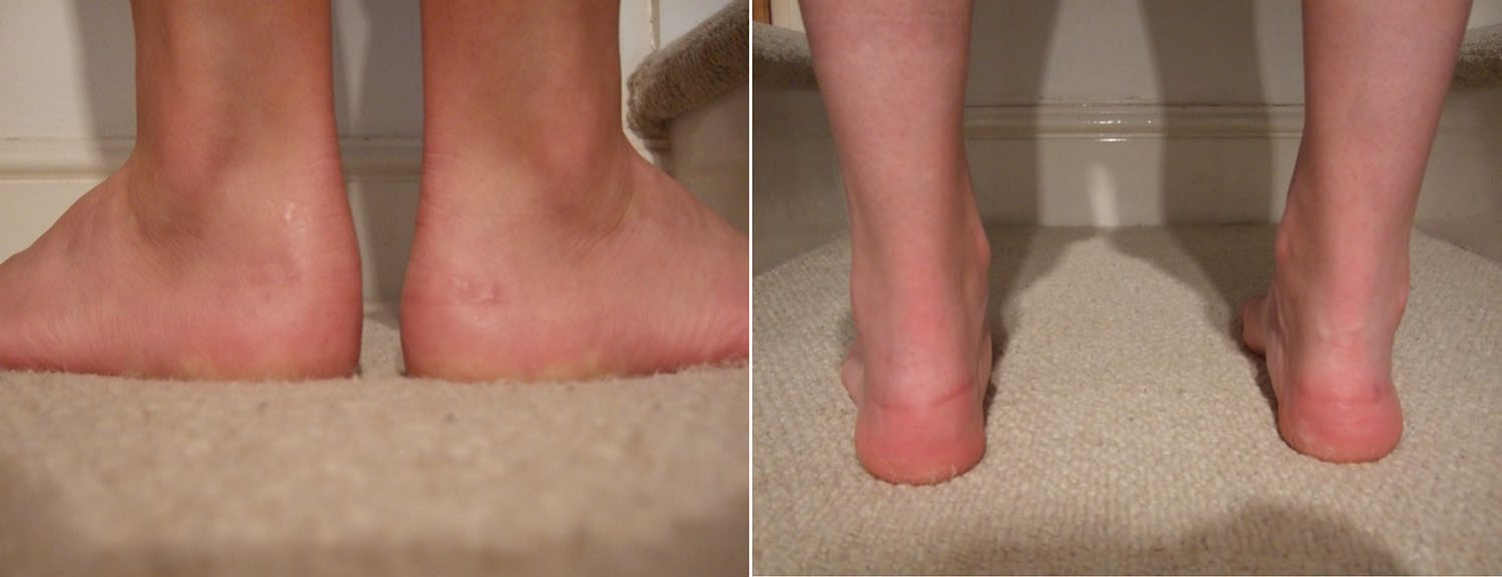
Failed lateral endoscopic approach revised with Cincinnati (right: pre-operative, left: 7 months post-operatively).

The surgical interventions were performed by one experienced surgeon (NM) using the technique previously published in the setting of primary surgery for IAT^20^. Under general or spinal anesthesia, the patients were positioned prone. The Achilles tendon and calcaneal tuberosity were marked as anatomical landmarks. A calf tourniquet was inflated to 250 mmHg, after exsanguination. An open approach using a Cincinnati incision was performed in all patients (Figs. 1, 2). A 7 cm to 10 cm semi-circular transverse skin incision was made over the tuberosity of the calcaneus. The medial and lateral margins of the Achilles tendon were then exposed and separated from the subcutaneous bursa by blunt dissection, allowing full access to the Achilles tendon and posterosuperior corner of the calcaneus, avoiding to detach of the tendon. An osteotomy of the posterosuperior corner of the calcaneus was executed. Areas of visually identifiable tendon abnormalities were excised. Subcutaneous tissues and skin were sutured in a standard fashion. We advise against the use of drains^20^.

A below-knee weightbearing synthetic cast was applied with the foot plantigrade. Patients were discharged within eight hours post-operatively, using elbow crutches and wearing a standard plaster shoe. Weight-bearing on the operated leg was allowed as tolerated. Two weeks after the index procedure, the cast was removed, and patients were advised to wear a flat sandal or a flat shoe with an open back for another four weeks. After removal of the cast, stationary cycling was recommended and weightbearing as able encouraged. From the 6th to the 12th week, isometric contraction of the gastrocnemius-soleus complex and gentle concentric contraction of the calf muscles were recommended. Proprioception, active plantar flexion, inversion and eversion exercises were allowed against manual resistance provided by a physiotherapist. Gentle non weight bearing training was allowed after 6 weeks, with more vigorous exercises allowed after 12 weeks. After a further four weeks, non-weight bearing-training exercises was introduced (cross training on the elliptical machine, stepping on stepping machine). Jogging was gradually introduced starting from the sixth post-operative month.

### Outcomes of interest

The following data were collected: mean age, gender, number and approach of previous failed treatments. Patients were assessed pre-operatively, and at 3, 6, 12 and 24 months after the index procedure. The Victorian Institute of Sport Assessment Scale for Achilles Tendinopathy (VISA-A)^21^, the EQ5D questionnaire^22^ and the visual analogic scale (VAS)^23^ were performed. The EQ-5D is a PROM evaluating the general health for clinical and socioeconomic appraisal^24^. The version 3L (EQ-5D-3L) was used for the present study. The EQ-5D-3L is composed by 5 items: (1) mobility, (2) self-care, (3) usual activities, (4) pain/discomfort, and (5) anxiety/depression. Each item is evaluated as 1 (best score) to 3 (worst score). The EQ-5D also involves the Thermometer Scale, which allows to obtain a global score to generally quantify the quality of life of the patient from 0 (no pain) to 100 (very severe pain). The VAS is a 0–100 pain rating system very similar to the EQ-5D Thermometer Scale, but it focuses exclusively on the pain perceived by the patient, not on the overall quality of life. The VISA-A is a validated PROM that evaluates specifically Achilles tendinopathy in a 0 (high severity) to 100 (no severity) scale^25^. It consists of 10 items evaluating the self-reported perception of pain and the consequent limitation in performing different tasks (e.g. walking, single leg heel raises, etc.).

### Statistical analysis

All statistical analyses were performed by one author (FM). The IBM SPSS software was used for the analysis. To evaluate the improvement of PROMs form baseline to the last follow-up, the mean difference (MD) and the paired t-test were used. Values of *P* < 0.05 considered statistically significant. For the analysis of the improvement of PROMs at the various follow-up appointments (from baseline to 3, from 3 to 6, from 6 to 12 and from 12 to 24 months), the P values were corrected using the Repeated Measure Analysis Of Variance (RM-ANOVA) followed by Bonferroni *post-hoc* test.

### Ethical approval

This study complies with ethical standards and was approved by the Ethical Committee of the University of Salerno (CESa 01,252,009/Rev2).

### Consent to Participate

All patients gave informed consent to participate.

### Consent to publish

All patients gave informed consent to publish their data.

## Results

### Recruitment process

**Figure 3:**
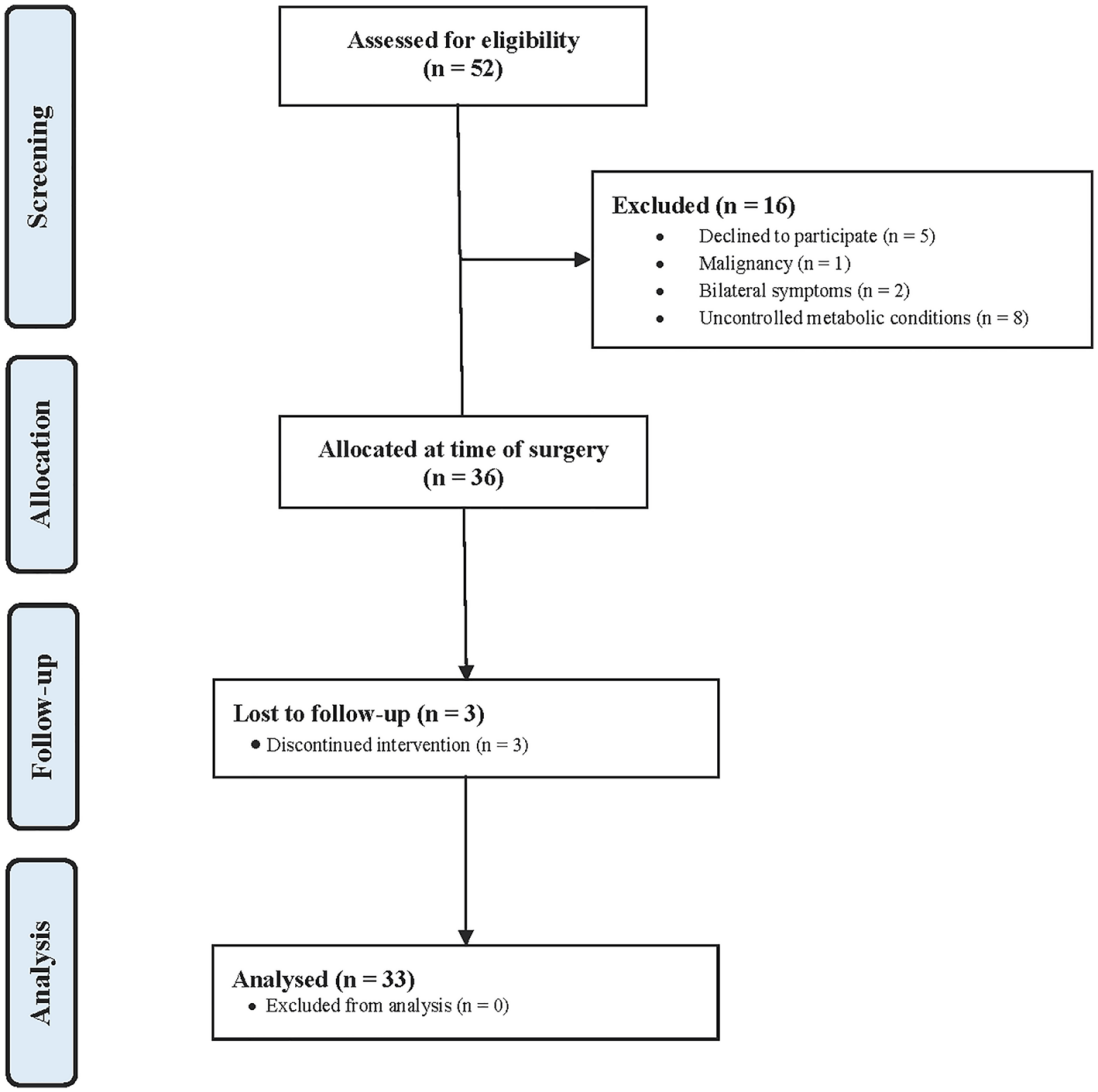
Flow-chart of the enrolment process.

A total of 52 patients underwent the index procedure. Patients were not eligible for the present study because of the following reasons: uncontrolled chronic disease and/ or blood test abnormalities (*N* = 8); malignancy (*N* = 1); bilateral insertional tendinopathy (*N* = 2); five patients did not wish to participate in the study. Finally, 36 patients who underwent the index surgery were recruited for the purposed of the present study. Of these, three patients were lost to follow-up. Eventually, 33 patients were included in the present study. The flow-chart of the enrolment process is shown in Fig. 3.

### Patient demographics

The mean age of the patients was 43.9 ± 10.6 years. 27% (9 of 33 patients) were female. The left side was involved in 58% (19 of 33) of patients. Following to the primary surgical procedure, once the symptoms had not improved, 45.5% (15 of 33) of patients had undergone one other cycle of conservative management (including rest, anti-inflammatory medications, physical and shockwave therapy, orthosis, immobilization), 24.2% (8 of 33) had two, 15.2% (5 of 33) had three, 12.1% (4 of 33) had four and 3.0% (1 of 33) had six. Table 1 showed in detail the demographic data of the included patients.

**Table 1:** Demographic data of the included patients.

Demographic at baseline	
Patients	33
Age	43.9 ± 10.6
Female	27% (9/33)
Left side	58% (19/33)
**Previous interventions**	
1	45.5% (15/33)
2	24.2% (8/33)
3	15.2% (5/33)
4	12.1% (4/33)
5	0.0% (0/33)
6	3.0% (1/33)

### Outcomes of interest

No clinically relevant complications were reported in any of the patients. Most of subscales of EQ5D improved form baseline to last follow-up: Usual Activities (MD -0.6; *P* = 0.01), Mobility (MD − 0.6; *P* = 0.03), Pain/ Discomfort (MD − 0.7; *P* = 0.001), Thermometer (MD 13.3; *P* = 0.04). No statistically significant change for the subscales Self-Care (*P* = 0.08) and Anxiety-Depression (*P* = 0.1) was evidenced. Table 2 displays the improvement of the EQ5D score at the various follow-up appointments.

**Table 2:** Improvement of the EQ5D at the various follow-up appointments (*statistically significant following Bonferroni *post-hoc* correction).

Endpoint	Values					Mean difference			
	Pre-operatively	3-month	6-month	12-month	24-month	Pre-operatively	3–6 months	6–12 months	12–24 months
EQ5D usual activities	1.9 ± 0.5	1.4 ± 0.5	1.4 ± 0.6	1.2 ± 0.5	1.3 ± 0.6	− 0.53 (*P* = 0.01)*	0.02 (*P* = 0.5)	− 0.20 (*P* = 0.1)	0.07 (*P* = 0.3)
EQ5D anxiety-depression	1.4 ± 0.5	1.2 ± 0.4	1.1 ± 0.3	1.2 ± 0.4	1.2 ± 0.4	− 0.21 (*P* = 0.04)	0.09 (*P* = 0.3)	0.06 (*P* = 0.8)	0.01 (*P* = 0.9)
EQ5D mobility	1.8 ± 0.4	1.4 ± 0.5	1.3 ± 0.5	1.2 ± 0.4	1.2 ± 0.4	− 0.43 (*P* = 0.009)	− 0.14 (*P* = 0.03)*	− 0.04 (*P* = 0.6)	0.01 (*P* = 0.9)
EQ5D pain/discomfort	2.1 ± 0.5	1.7 ± 0.6	1.5 ± 0.5	1.4 ± 0.5	1.5 ± 0.5	− 0.40 (*P* = 0.01)*	− 0.21 (*P* = 0.08)	− 0.14 (*P* = 0.04)	0.08 (*P* = 0.6)
EQ5D self-care	1.1 ± 0.4	1.1 ± 0.3	1.0 ± 0.0	1.0 ± 0.0	1.0 ± 0.0	− 0.07 (*P* = 0.8)	− 0.07 (*P* = 0.7)	− 0.00 (*P* = 0.9)	− 0.00 (*P* = 0.9)
EQ5D thermometer scale	62.1 ± 25.1	75.8 ± 23.6	80.8 ± 18.1	82.6 ± 15.5	75.4 ± 18.2	13.77 (*P* < 0.0001)*	5.01(*P* = 0.002)*	1.86 (*P* = 0.09)	− 7.2 (*P* = 0.06)

The VISA-A score improved significantly at last follow-up (MD 34.8; *P* < 0.0001), as did the VAS score (MD − 50.8; *P* < 0.0001). Table 3 shows the improvement of the VISA-A and VAS scores at the various follow-up appointments.

**Table 3:** Improvement of the VISA-A and VAS scores at the various follow-up appointments (*statistically significant following Bonferroni *post-hoc* correction).

Endpoint	Values					Mean difference			
	Pre-operatively	3-month	6-month	12-month	24-month	Pre-3 months	3–6 months	6–12 months	12–24 months
VISA-A (%)	39.7 ± 23	59.8 ± 25.9	66.6 ± 25.4	77.8 ± 25.3	74.5 ± 29.1	20.09 (P = 0.0001)*	6.80 (*P* = 0.09)	11.18 (*P* = 0.02)*	− -.24 (*P* = 0.1)
VAS (%)	64.4 ± 19.0	30.4 ± 28.6	21.3 ± 19.3	15.0 ± 21.0	13.6 ± 20.4	− 33.99 (*P* = 0.003)*	− 9.18 (*P* = 0.06)	− 6.26 (*P* = 0.2)	− 1.41 (*P* = 0.4)

## Discussion

The most important finding of the present study was that a transverse Cincinnati incision followed by debridement and osteotomy of the posterosuperior corner of the calcaneus as revision surgery for IAT achieves clinically relevant improvement in the VISA-A and VAS at 24 months follow-up. A statistically significant improvement of the EQD5 subscales Usual Activities, Mobility, Pain/Discomfort and Thermometer was also observed. The EQD5 subscale Anxiety-Depression remained unchanged during the whole follow-up. No clinically relevant complications were experienced.

Patients with a history of failed surgical treatments for insertional IAT are challenging, and no conservative or surgical modalities can guarantee symptoms resolution for IAT. Several surgical and non-surgical treatments have been described, but no consensus has been reached regarding the most effective modality^26,27^. Given the minimal postoperative discomfort for the patients, in our hands the Cincinnati incision has become the preferred approach in past few years^5^. Curvilinear, midline and the lateral longitudinal incisions are alternatives to the Cincinnati transverse incision^4,28^, but are prone to complications of wound healing and breakdown and iatrogenic nerve injury^29^. The Cincinnati approach allows adequate exposure of the distal Achilles tendon and the posterior aspect of the calcaneous, enabling debridement of the pathological tissue and of the subcutaneous bursa^20^. In comparison to medial and/or lateral incisions, the Cincinnati incision allows full exposure of both the medial and lateral aspects of the insertion of the AT on the calcaneus. This may be a reason for its success, as medial and lateral approaches may offer limited exposure, not allowing adequate debridement. Furthermore, longitudinal posterior AT splitting approaches may produce hypertrophic scars and AT adherences, resulting in unfavourable outcomes. As the Cincinnati incision for the purposes of surgery on the insertion of the Achilles tendon is centred over the calcaneal tuberosity and does not extend anteriorly as much as it would be required if club foot surgery were to be undertaken, the chances of nerve damage are minimised, and, as demonstrated by the present and previous studies, resolve spontaneously if and when they occur^20^. It is theoretically possible to encounter some of the branches of the sural and posterior tibial nerves. This would be indeed the case when facing patients with a club foot, where the incision is extended much more anteriorly, both medially and laterally, than what is required when dealing with insertional Achilles tendinopathy. Indeed, one of our patients developed anaesthesia/hypoesthesia laterally, distal to the incision. One patient reported a patchy sensation of paraesthesia medially and distally to the incision on removal of the plaster cast, but this resolved by the next appointment, four weeks following the index procedure.

Tendons have limited regenerative ability^30^. The process of tendon healing is complex, with intrinsic and extrinsic pathways, that are still not fully clarified^31^. The tendon healing process results in a fibrotic scar with reduced elasticity, strength and mechanical efficiency compared to the native tissue^32–35^. Post-operative soft tissue adherences are also common^36,37^. Histologically, disorganised vascularity, a haphazard cellular response, and disordered metabolic activity are commonly observed in tendinopathies^38,39^. These features are related with a greater risk of recalcitrant tendinopathy^36,40,41^. In this perspective, often planning to return to sport in few months following surgery is not realistic. Although in some patients the time to return to sport may take longer, 8–10 months of rehabilitations should be expected even after well performed surgery. This long period of time often leads to frustration in continuing the rehabilitation program, and early attempts to return to sport represent a common cause of failure of technically well performed surgery^42,43^. Moreover, even if combined with the best physiotherapy care, success cannot be guaranteed^20^. Patients must be aware of these issues, and must be strictly followed by their operating physician and physiotherapist. Rehabilitation aims to gradually increase patient’s activity levels^44^. Swimming, rowing, cycling, elliptical training and stepping machine training are strongly recommended to recover muscle strength and endurance. After the first six months, impact activities are allowed^45^. Also, it should be noted that even extensive revision surgery cannot guarantee return to normality: patients should be made aware of this, and their unreasonable expectations must be mitigated.

This study has some limitations. No patients with metabolic disorders were enrolled in the present study: we are aware of the association between dysmetabolic diseases and insertional Achilles tendinopathy^46^. Also, we did not collect data on smoking. Since patients with failure of previous surgery for insertional tendinopathy of the Achilles tendon are uncommon in clinical practice, the number of procedures available for analysis was limited. This may have affected the capability to identify possible variables leading to poor outcomes. Therefore, even if methodologically well conducted, results from the present investigation must be carefully interpreted, and future studies with greater sample size are necessary. The unblinded nature of the study design along with the lack of randomization are also important limitations. However, randomisation in revision surgery may not be easily acceptable by the patients and the surgeons alike. A randomised controlled trial comparing two or more techniques could be planned to demonstrate which one offers superior outcomes. The patients enrolled in the present investigation were all secondary and tertiary referrals, and not all were able to describe all the treatments which they were subjected before deciding to undergo further surgery; also, hospital notes from the other centres were often not available. However, we point out that the number of patients reported in the present study is comparable to many of the studies on primary surgery of the condition at hand, and that, with the numbers available, the results obtained are clinically relevant and statistically significant. Clearly, this is a single centre single surgeon study, and the operating surgeon has a special interest in these injuries. These results need therefore to be validated by larger multicentre studies. A formal power analysis was not performed: the number of patients to enrol in the study was nevertheless relatively large considering how uncommon these patients are. However, despite this partial weakness of the present investigation, our selection and recruitment process, our assessment criteria and our follow up were extremely rigorous, and performed in strict scientific fashion. Also, with the numbers of patients enrolled, the results of our study are univocal. These limitations may affect the reliability of our conclusion; therefore, data must be interpreted with caution. However, despite these limitations, all the surgical procedures were performed in the same fashion and with same instruments, modalities and materials. All the surgeries were performed by the same fellowship trained surgeon with long surgical experience and scientific expertise on the field of AT surgery. Given the limited study population size, a formal investigation of prognostic factor which may influence the surgical outcome was not conducted, and thereby further investigations involving larger population are required.

## Conclusion

The management of patients with IAT in whom symptoms have not abated following surgery is problematic, and evidence concerning the management and outcomes of those patients are lacking. A transverse posterior heel Cincinnati incision, followed by accurate tendon and bursae debridement and a wide osteotomy of the posterosuperior corner of the calcaneus as revision surgery for IAT demonstrated feasibility and reliability, achieving clinically relevant improvement in the VISA-A, EQ5D and VAS at an average of 24 months follow-up.

## Data Availability

The datasets generated during and/or analysed during the current study are available throughout the manuscript.
